# Association between Height-Changing Scores and Risk of Sarcopenia Estimated from Anthropometric Measurements in Older Adults: A Cross-Sectional Study

**DOI:** 10.3390/healthcare12101005

**Published:** 2024-05-13

**Authors:** Siwaluk Srikrajang, Narucha Komolsuradej

**Affiliations:** 1 Department of Physical Therapy, Faculty of Medicine, Prince of Songkla University, 15 Kanchanavanich Road, Songkhla 90110, Thailand; siwaluk.s@psu.ac.th; 2 Department of Family and Preventive Medicine, Faculty of Medicine, Prince of Songkla University, 15 Kanchanavanich Road, Songkhla 90110, Thailand

**Keywords:** anthropometry, elderly, height loss, sarcopenia

## Abstract

Anthropometric assessments are commonly used to diagnose sarcopenia in older adults. However, the ongoing exploration of novel approaches aims to improve the early detection of sarcopenia. This study investigated the association between the height-changing score (HCS) and the risk of sarcopenia defined by anthropometric measurements in 340 older adults (mean age: 66.2 years). The HCS derived from the difference in height and demi-span equivalent height (DEH) was used as an indicator of declining height in the older adults. Multivariate logistic regression analysis revealed a significant association between the HCS and the risk of sarcopenia in both male and female older adults (OR = 1.146, 95% CI [1.021, 1.286], *p* = 0.021). In addition, income, BMI, and nutritional status were significantly associated with the risk of sarcopenia (OR = −1.933, 95% CI [0.271, 0.986], *p* = 0.045; OR = −2.099, 95% CI [0.386, 0.587], *p* < 0.001; OR = −1.443, 95% CI [0.555, 0.866], *p* = 0.001, respectively). The white blood cell count, lymphocyte count, and HDL cholesterol were blood biomarkers significantly correlated with calf circumference. It can be suggested that the HCS acts as an indicator and screening tool for sarcopenia risk in older adults, highlighting the potential impact of decreased height on muscle mass loss. Encouraging nutritional support can help mitigate the risk of sarcopenia.

## 1. Introduction

According to the World Health Organization (WHO), there is a noticeable increase in the number of people aged over 60. This prediction shows the increase in the aging population, which represents a significant increase to 1.4 billion by 2030 [1]. Aging causes a variety of physiological changes, with one of the most common and important of those being the gradual loss of muscle mass, strength, and function due to age: also known as “sarcopenia”. It was previously estimated that sarcopenia affects 10–16% of the world’s elderly, but this proportion has increased, ranging from 18% to 66% for elderly patients [2]. Sarcopenia is a disorder that poses significant threats to the overall well-being, as well as mobility restrictions, functional independence, and poor quality of life of older adults [3,4,5].

Diagnostic measurements of sarcopenia have traditionally focused on examining muscle mass and strength [6]. Anthropometric measurements have been studied and proposed to be used to screen for the risk of sarcopenia because of their ease of protocol testing, as well as their being non-expensive, non-invasive, and practical in clinical settings. Several studies have confidently concluded that the calf and mid-arm circumference are anthropometric measurements that can diagnose sarcopenia and indicate muscle mass loss in the general and elderly population [7,8]. Calf circumference has been reported to be positively correlated with bioelectrical impedance (sensitivity 71.43 and specificity 72.09) and DXA measurements (sensitivity 91%; specificity 84%) [8], hand grip strength (r = 0.84), and low walking speed [9]. In addition, the mid-arm circumference has been reported to be strongly correlated with the muscle mass index (r = 0.698–0.726), and the area under the receiver operating characteristic curve is 0.86, indicating low muscle mass [7]. However, the complex relationship of the components affecting musculoskeletal health needs a more comprehensive approach. Both theoretical and clinical studies have suggested that muscles and skeletal health interact with each other [10,11] due to specific growth factors, potentially mediating the phenomenon of bidirectional muscle–bone crosstalk [12,13]. Height decline occurs from middle age (30 to 50 years old) and continues to decease with accelerated rates by age [14,15]. The stature height decline in the elderly decreases at a rate between 0.08% and 0.10% per year for men and between 0.12% and 0.14% for women, resulting in a 2 cm to 4 cm drop in height over a lifetime [16]. Several studies have suggested that measuring height is not a precise test of estimating maximum height in the elderly due to age-related height decrease [9,15]. Although many anthropometric measurements have been used to calculate this, some studies have suggested that among the differences in anthropometric measurements, demi-span provides a highly accurate estimate of the maximum standing height, generally reaching about 30 years old, and does not significantly decrease with age compared to the overall height [16,17]. Therefore, in the case of stature height being less than the demi-span equivalent height (DEH), it could be hypothesized that height declines in older adults. The difference between DEH and stature height has been stated in a previous study in that it estimates the correlation between DEH and stature height [17]. However, these values have not been used to detect any musculoskeletal health problems, even if the study does delve into the biomechanical complexities of height loss.

Previous studies have reported an association between height loss and an increased risk of sarcopenia [12,13]. Although it is well known that muscle and skeletal health requires adequate care and nutritional support, current anthropometric measurements can only detect either height change or sarcopenia independently. Therefore, there is a gap in the literature on assessment tools that can evaluate both the risk of sarcopenia and height loss simultaneously. Based on previous discussions, the difference between DEH and stature height could be an indicator of height decline, which could potentially reflect muscle mass loss due to associations between bone and muscle.

In this context, this study aimed to investigate the possible association between the height changing score (HCS; difference between DEH and stature height) and sarcopenia risk, which is diagnosed by defining muscle mass by the calf and mid-arm circumference in older adults. This study aimed to provide a new perspective that could enhance the use of HCS for the early detection of sarcopenia. This is ultimately aimed to promote more targeted interventions for the aging population and to define the importance of preventing bone degeneration and muscle mass loss in the elderly simultaneously.

## 2. Materials and Methods

### 2.1. Participants

**Figure 1:**
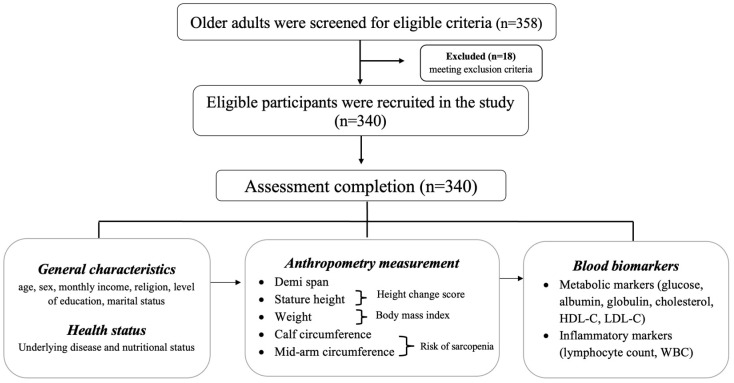
Study flow of the participants (n = 340).

This cross-sectional study was conducted at Songklanagarind Hospital, Thailand. Participants included 340 older adults (205 females and 135 males) (age mean ± SD: 66.2 ± 4.1 years) and participants were recruited from 15 February 2021 to 31 July 2021 through purposive sampling from the general practitioner (GP) and Primary Care Unit (PCU). Eligible participants were over 60 years of age who visited the GP and PCU clinics for follow-up of non-communicable diseases (diabetes, dyslipidemia, hypertension, obstructive pulmonary disease, gout, and coronary artery disease). Exclusion criteria included physical impairment that could hinder anthropometry assessment (disabilities, limb amputation, bone deformity, scoliosis, and thoracic hyperkyphotic), history of fracture (leg bones, arm bones, or spine) as well as emergent infectious status that could affect hematological characteristics. The flow of participants is shown in Figure 1.

### 2.2. Data Collection

After complete screening and signing of informed consent, participants were transferred to a private laboratory for data collection. Sociodemographic characteristics were collected through face-to-face interviews using standard questionnaires including age, gender, religion, education, income, and underlying disease. Thereafter, anthropometric measurements were performed using the same examiners with good intra-rater reliability (ICC 0.88–0.94) including body weight, height, demi-span, calf circumference, and mid-arm circumference through standard protocols. The HCS was derived from “height changing equation” which was estimated by subtracting the stature height from DEH, which was calculated from the Bassey equation [17,18,19] as follow.

HCS for females: [(1.35 × demi-span in cm) + 60.0] − stature height

HCS for males: [(1.40 × demi-span in cm) + 57.8] − stature height

### 2.3. Measurement

For body weighing, participants were instructed to wear minimal light clothing and stand barefoot on an electronic scale that was 0.1 kg accurate (Nagata model: BW3323MH, Nagata Scale Co., Ltd., Taiwan, China). The scale was calibrated daily using standard protocols before use in the study.

The height measurements were made using a standard stadiometer to use the nearest millimeter. Participants were required to take off their shoes and stand against a wall, with their heads facing forward in a Frankfurt plane. After completing the weight and height measurements, the weight (kg) and height (m) were squared and converted to BMI. 

Demi-span measurements included participants standing against a wall with shoulder abduction at 90 degrees from the anatomical position. The measurement from the midpoint of the suprasternal notch to the metacarpophalangeal joint of the third and fourth fingers was measured using a measuring tape, with a precision of 0.1 cm. Mid-arm circumference was measured while participants sat comfortably, with arms relaxed and removing part of their clothing which covered the upper arm area, with relaxed muscles. The midpoint between the shoulder (acromion process) and the elbow (olecranon process) on the upper arm was then marked. The flexible and non-stretchable tape was wrapped around the arm at the identified midpoint. It was ensured that the tape was snug, but not tight, without compressing the tissues. Measurements were taken in centimeters, with an accuracy of 0.1 cm. Calf circumference was measured in the standing position to prevent overestimation. Measurements were conducted at the widest path of the right leg calf, which was suggested as having a better diagnostic performance [20]. Then, a flexible measuring tape was wrapped around the identified point, without compressing the calf tissues. The measurement was recorded in centimeters, with an accuracy of 0.01 cm.

Risk of sarcopenia was assessed from anthropometric measurements: calf circumference and mid-arm circumference. Participants were assigned to risk in the sarcopenia group if they met both of the following criteria.

1. Calf circumference less than 34 cm for men and 33 cm for women [20].
2. A mid-arm circumference of ≤28.6 cm for men and ≤27.5 cm for women [7].

After completing the anthropometry measurements, the laboratory results of blood biomarkers were retrieved from the hospital information system (HIS). The metabolism biomarkers (including albumin, globulin, cholesterol, HDL-C, and LDL-C) and inflammatory biomarkers (including WBC and lymphocyte count) were recorded for analysis. 

### 2.4. Statistical Analysis

Double entry data were recorded in Epidata version 3.1 (Comprehensive Data Management and Basic Statistical Analysis System, Odense, Denmark, 2008). Statistical analyses were performed using R Studio version 3.3 (Public Benefit Corporation, Boston, MA, USA, 2009). Demographic data are presented as percentages, means (standard deviations), and median (interquartile range). The associations between HCS and sarcopenia risk were assessed using Pearson’s product moment correlation and Spearman’s rank correlation. The associations between sarcopenia risk group and related factors were analyzed using independent sample *t*-tests, Mann–Whitney U tests, chi-square tests, and Fisher’s exact tests. Strong association factors of sarcopenia risk defined by anthropometry were determined by multivariate logistic regression analyses. Spearman’s rank correlation was used to evaluate the correlations among blood biomarkers. The significance level was set to *p* < 0.05.

## 3. Results

### 3.1. Characteristics of the Participants

**Figure 2:**
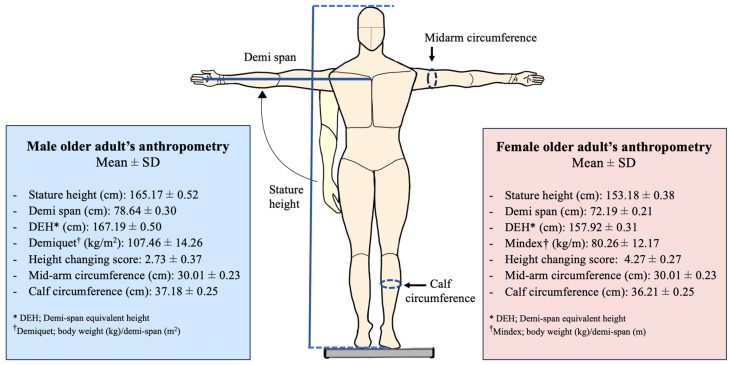
Anthropometry measurements (including: stature height, demi-span, demi-span estimated height, Demiquet, Mindex, HCSs, mid-arm circumference, and calf circumference) in older male and female adults (n = 340).

A total of 340 older adults were recruited for this study. Most of the participants were female (60.3%), with a mean (SD) age of 66.2 (4.1) years. The mean BMI of participants was 26.28 (3.01) kg/m^2^, which could be classified as overweight. According to the cut-off scores of the calf and mid-arm circumference, most of the older adults had no risk of sarcopenia (81.18%). This study found that the HCS presented with significantly higher values in older female adults (*p* = 0.001) (Figure 2). This study revealed that the age, education level, income, and MNA score were significantly different between older adults with and without risk of sarcopenia (*p* < 0.005), as well as anthropometric-related factors, including BMI and HCS (Table 1). 

### 3.2. Association between HCS and Anthropometric Measurement

**Figure 3:**
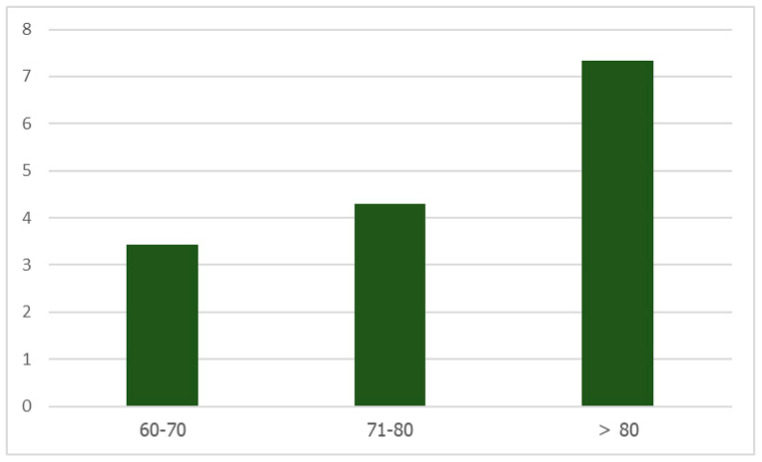
Bar graph presenting the HCS in different age ranges (60–70, 71–80, and >80 years).

As defined by the anthropometric measurement, there was a significantly negative correlation between the HCS and calf circumference, as well as the risk of sarcopenia (Table 2). As evident, this phenomenon was present in both male and female older adults. Most participants had a positive HCS value (85%). Furthermore, this study found that the HCS increased with age (Figure 3) and was found to be higher in older female adults (Figure 2). The results from the multivariate logistic regression analysis (Model 1) revealed that the HCS was significantly positively associated with the risk of sarcopenia (OR = 1.137, 95%CI [1.016, 1.274], *p* = 0.025) (Table 3). Furthermore, lower income levels (OR = 1.933, 95% CI [0.271, 0.986], *p* = 0.045) and a lower BMI (OR = 0.454, 95% CI [0.369, 0.559] *p* < 0.001) were significantly associated with a higher risk of sarcopenia. In addition, Model 2 also showed that low MNA scores were significantly positively associated with the risk of sarcopenia (OR = 1.443, 95% CI [1.555, 0.866] *p* = 0.001).

**Figure 4:**
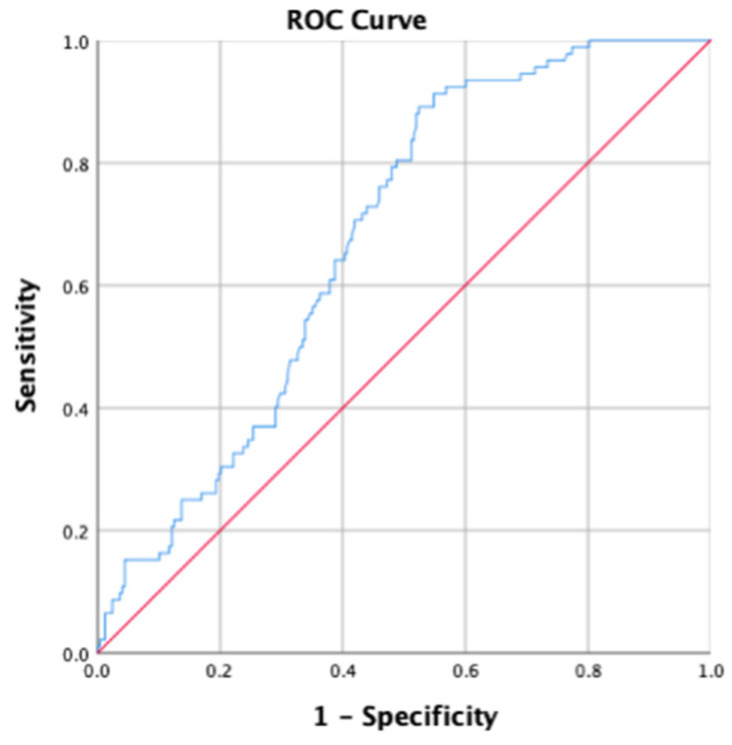
The area under the ROC curve demonstrates the efficacy of HCS in identifying risk of sarcopenia among older adults.

Figure 4 shows the potential for the use of HCS in screening the risk for sarcopenia in the elderly, with an area under the ROC curve of 0.691. In this study, the cut-off level was determined at 2.1 cm. 

In this study, the globulin level, calf circumference, and mid-arm circumference were excluded from the regression model to mitigate collinearity with the albumin level, nutritional status, and risk of sarcopenia, respectively.

### 3.3. Blood Biomarkers and Risk of Sarcopenia Defined by Anthropometry Measurements

In this study, only HDL-C was the metabolic biomarker that showed a correlation with anthropometric measurements (*p* < 0.05). The inflammatory markers investigated in this study, including the lymphocyte count and WBC, showed a strong correlation with the calf and mid-arm circumference. In addition, this study found that the calf and mid-arm circumference showed a similar pattern of correlations to blood biomarkers, as seen in Table 4.

## 4. Discussion

This study investigated the association between the “height changing score” and the risk of sarcopenia in the older adults, suggesting the possibility of using the height changing equation as an anthropometric measurement for screening for sarcopenia. This study demonstrated that the HCS derived from the height changing equation (difference between DEH and stature height) was positively associated with an increased risk of sarcopenia in older adults. Furthermore, this study demonstrated that lower income, BMI, and MNA scores were associated with the risk of sarcopenia. The current study also investigated the possibility of using the HCS as an anthropometric measurement for screening for sarcopenia and attempted to identify the cut-off value through the area under the ROC curve. Consequently, the AUC showed that this was an acceptable tool to differentiate older adults with or without a risk of sarcopenia (AUC > 0.6), with a cut-off level of 2.1 cm [21].

In this study, most older adults had a positive HCS value (85%), which can be estimated to be a decrease in height with age. This is because the values were derived from the DEH (the Bassey equations), which was reported as the most precise measurement predicting the maximum height. This is because the length of the arm bone does not change with age. Furthermore, this study found that the HCS increased with age, especially in older adults over 80 years of age, as shown in Figure 3. This phenomenon is also supported by previous studies that reported that the mean values of height and BMI decreased with age [22,23]. The HCS showed a significant association with the calf circumference and risk of sarcopenia. However, there is no association between the HCS and the mid-arm circumference, even though both circumferences have a strong correlation with each other (ρ = 0.690, *p* < 0.001). It can be hypothesized that the calf circumference may be affected by musculoskeletal health deterioration rather than the mid-arm circumference of the elderly. This is because they are used more frequently in daily activities such as walking and running and use less upper limb muscle [24,25]. In a prior study, the calf circumference exhibited a higher rate of change. For elderly women, a one-unit increase in the calf circumference reduced the sarcopenia risk by approximately 72%, compared to a 16% reduction with the arm muscle area. This suggests that the calf circumference may be more sensitive to detecting sarcopenia [26]. However, the combination of the calf circumference and mid-arm circumference, used as the anchor anthropometric measurements in this study, could affect the outcome, potentially contributing to the lower AUC of the HCS in detecting the risk of sarcopenia.

The HCS used in this study was mentioned in a recent study which also indicated that the DEH exceeded the stature height of participants aged 65 years and older and increased with age, with the finding of a higher difference in older female adults [17]. This is probably explained by the mechanism of height reduction in the elderly from physical changes. Recent studies have highlighted the relationship between height loss and the risk of sarcopenia using various protocols, including subtracting the currently measured height from the highest recalled height or examining the rate of height change over a 2-year period. Despite the different protocols, the association between height loss and sarcopenia risk has consistently shown strong correlations, with odds ratios ranging from 2.30 to 2.67 [14,26]. Similarly, our study found a positive association between the HCS and the risk of sarcopenia, with a lower odds ratio of 1.146. This difference in the odds ratio can be attributed to previous studies using more accurate diagnostic tools such as grip strength and walking speed to assess the risk of sarcopenia. However, a previous protocol for detecting height loss could not be proposed as an assessment tool to detect sarcopenia like the HCS in this study.

The relationship between declining height and sarcopenia has been stated behind the mechanism that height loss in older adults is linked with a low bone density, which can represent age-related degenerative musculoskeletal health [26,27,28] such as an increasing rate of osteoporosis [29], thoracic kyphoscoliosis [13], vertebral fracture [30], frailty, falling, and sarcopenia [26]. Several factors have been reported as risk factors of height loss when age is increasing, such as post-menopausal calcium and vitamin D deficiencies [9], the use of corticosteroids, a lack of physical activity [31], poor nutritional status [32], and a deterioration in muscle and tissue components [33]. The positive association between the HCS and the risk of sarcopenia found in this study could be supporting the fact that declining height and muscle mass can occur together. 

In addition, this study found that a low income, low MNA scores, high levels of HDL-C, and inflammatory biomarkers were significantly associated with a higher risk of sarcopenia. Low-income individuals may have difficulty in making healthy lifestyle choices, including an adequate protein intake, and physical activity may be reduced due to limited access to medical resources, such as fitness facilities or recreational spaces. This can also include chronic stress, which can lead to inflammation and muscle breakdown. Moreover, social determinants of health, such as education, employment, and housing, may also play a role in this. This current study found significantly lower MNA scores in participants with a risk of sarcopenia. Notably, the higher rate of an abnormal nutritional status seems to be found in older adults that have a positive HCS. Moreover, the larger HCS seems to increase with an age greater than 70 in men and 65 in women. This result is consistent with previous studies that indicate that the height loss increases with age [14,34]. It is well known that resolving the abnormal nutritional status through a balanced diet, nutritional supplements, especially protein, vitamin D, antioxidant nutrients, and long-chain polyunsaturated fatty acids, combined with proper hydration is also important in preventing and managing sarcopenia [32]. Furthermore, the results of this current study are consistent with a previous study that demonstrated an inverse association between muscle mass markers and HDL-C [34] under the mechanism of a larger calf circumference being related to higher insulin resistance. This can cause an increased level of lipoprotein lipase, leading to a decrease in HDL-C [35]. Finally, a positive association between inflammatory biomarkers and anthropometric measurements was suggested in this study, but we could not assume this with confidence, as participants in this study had a normal range of WBCs and lymphocyte count and patients with emergency infection were excluded. 

The strength of this study was to investigate the HCS, a novel tool that can be used as a simple evaluation tool in clinical settings to detect both height loss and sarcopenia simultaneously. This capability could enable healthcare providers to develop more customized treatment plans for the elderly and design targeted nutritional support strategies. Furthermore, the study explored the relationship between anthropometry and blood biomarkers in the elderly, an area that has not been extensively studied before.

This study has some limitations. First, the diagnostic protocol for sarcopenia risk used only included anthropometry measurements (the calf and mid-arm circumference), which may have less of an ability for the detection of sarcopenia and produce a lower AUC of the HCS. Further studies are needed to investigate the correlation of the HCS with more precise assessment tools for sarcopenia, such as body composition and a physical function test [36]. Second, because of the cross-sectional design of this study, it cannot be confidently concluded that the HCS can accurately estimate height loss. To overcome these shortcomings, further studies are needed to investigate the HCS with height changes in retrospective or prospective cohort designs.

## 5. Conclusions

The HCS calculated by subtracting the stature height from DEH shows a strong correlation with the risk of sarcopenia assessed by anthropometric measurements in the elderly. This study indicates the potential applicability of the “height changing equation” as an alternative measure for screening the risk of sarcopenia in the geriatric healthcare setting. In addition, a low income and abnormal nutritional status appear to be correlated with sarcopenia. This study supports the importance of nutritional support, especially from protein and calcium-rich sources, for the simultaneous maintenance of muscle and bone health in older adults.

## Figures and Tables

**Table 1:** Characteristics of 340 older Thai adults, according to sarcopenia risk screened by calf circumference and mid-arm circumference.

Factors	Sarcopenia Risk		*p*-Value
	Risk of Sarcopenia n (%) Overall = 64	No Risk of Sarcopenia n (%) Overall = 276	
Gender			
- Female (n = 205)	44 (68.75)	161 (58.33)	0.081 ^f^
- Male (n = 135)	20 (31.25)	115 (41.67)	
Age (years) Mean (SD)	69.1 (6.4)	65.8 (4.8)	0.001 *
Religion			
- Buddhism	57 (89.06)	260 (94.20)	0.320 ^c^
- Other	7 (10.93)	16 (5.79)	
Marital status			
- Married	49 (76.56)	204 (73.91)	0.619 ^c^
- Other	15 (23.44)	72 (26.09)	
Education level - None/under primary education/primary school - Secondary education level - Bachelor’s degree or higher level	29 (45.31) 5 (7.82) 30 (46.87)	59 (21.34) 61 (22.1) 156 (56.52)	0.003 *
Income (Thai Baht per month)			
- <10,000 (less than 281 USD)	31 (48.43)	74 (26.81)	0.002 *
- 10,000–30,000 (281–843 USD)	21 (32.82)	99 (35.86)	
- >30,000 (more than 843 USD)	12 (18.75)	103 (37.32)	
Underlying disease			
- DM	7 (10.94)	46 (16.57)	0.198 ^f^
- Hypertension	21 (32.81)	115 (41.67)	0.163 ^f^
- Dyslipidemia	58 (90.63)	254 (92.03)	0.442 ^f^
- Other (stroke, gout, COPD, myocardial infarction)	4 (6.25)	16 (5.79)	0.817 ^f^
Body Mass Index (kg/m^2^) [mean (SD)]	20.97 (0.25)	25.35 (0.18)	<0.001 *
MNA score			
- Risk of malnutrition	21 (32.81)	11 (4.19)	<0.001 ^f^
- Normal nutritional status	43 (67.19)	265 (96.01)	
Height-changing score			
[mean (SD)]			
- Female			
● Overall 60+	3.98 (0.29)	5.34 (0.36)	0.037 *
● 60–65	3.49 (0.39)	4.19 (0.92)	
● 65–69	4.25 (0.57)	7.75 (1.35)	
● 70–75	4.44 (0.98)	5.15 (0.84)	
● >75	4.65 (1.89)	6.91 (1.13)	
- Male			
● Overall 60+	2.33 (0.41)	4.59 (0.72)	0.019 *
● 60–65	1.96 (0.50)	3.40 (1.89)	
● 65–69	2.88 (0.88)	4.36 (0.96)	
● 70–75	2.64 (1.59)	4.13 (1.77)	
● >75	3.25 (1.66)	5.13 (1.53)	
Glucose (g%)	102.70 (1.19)	102.19 (2.65)	0.848 ^t^
Albumin (g%)	4.48 (0.05)	4.41 (0.11)	0.303 ^t^
Globulin (g%)	3.39 (0.24)	3.23 (0.16)	0.673 ^t^
Lymphocyte count (per µL)	2491.36 (117.80)	2051.26 (222.21)	0.007 *
Cholesterol (mg%)	178.05 (5.97)	205.14 (14.56)	0.817 ^t^
High-density lipoprotein (mg%)	54.14 (2.17)	59.13 (3.36)	0.788 ^t^
Low-density lipoprotein (mg%)	114.01 (5.22)	131.947 (14.08)	0.901 ^t^
WBC (cell/uL)	6631.78 (103.38)	6184.69 (202.03)	0.052 ^t^

* Significant difference between groups (*p* < 0.05). ^c^ Chi square test, ^f^ Fisher exact test, ^t^ *t*-test.

**Table 2:** The association of HCS, calf circumference, and mid-arm circumference in 135 male and 205 female older adults.

Sex of Participants	Mid-Arm Circumference ^†^	Calf Circumference ^†^	Risk of Sarcopenia ^‡^
Female (n = 205) Height-changing score	ρ = −0.016 *p* = 0.816	ρ = −0.201 *p* = 0.004 *	Pearson correlation = 0.147 *p* = 0.035 *
Male (n = 135) Height-changing score	ρ = −0.136 *p* = 0.115	ρ = −0.261 *p* = 0.002 *	Pearson correlation = 0.202 *p* = 0.019 *
Height-changing score (both male and female)	ρ = −0.073 *p* = 0.180	ρ = −0.233 *p* < 0.001 *	Pearson correlation = 0.154 *p* = 0.004 *

* Significant association (*p* < 0.05). ^†^ Spearman-rank correlation. ^‡^ Pearson’s product moment correlation.

**Table 3:** Results of the multivariate regression analysis assessing the associated factors of sarcopenia risk in 340 older adults.

	Risk of Sarcopenia			
	Odd Ratio	95% Confident Interval	Standard Error	*p*-Value
Model 1				
- Height-hanging score	1.137	1.016, 1.274	0.058	0.025
- Income	−1.933	0.271, 0.986	0.329	0.045
- BMI	−0.454	0.369, 0.559	0.106	<0.001
Model 2				
- Height-changing score	1.146	1.021, 1.286	0.059	0.021
- Income	−1.826	0.285, 1.051	0.333	0.070
- BMI	−2.099	0.386, 0.587	0.107	<0.001
- MNA score	−1.443	0.555, 0.866	0.113	0.001

Model 1: adjusted for gender, age, education level, lymphocyte count, and albumin level through stepwise regression analysis. Model 2: adjusted for gender, age, education level, lymphocyte count, albumin level, and nutritional status using stepwise regression analysis.

**Table 4:** The heatmap illustrates the correlation among blood biomarkers (metabolic and inflammatory markers), calf circumference, and mid-arm circumference in 340 older Thai adults.

Biomarkers	Lymphocyte Count	WBC	Albumin	Globulin	Cholesterol	HDL-C	LDL-C
calf circumference	ρ = 0.169	ρ = 0.144	ρ = 0.029	ρ = 0.037	ρ = −0.039	ρ = −0.131	ρ = 0.008
	*p* = 0.002 *	*p* = 0.008 *	*p* = 0.591	*p* = 0.788	*p* = 0.472	*p* = 0.016 *	*p* = 0.882
mid-arm circumference	ρ = 0.246	ρ = 0.259	ρ = 0.006	ρ = 0.124	ρ = −0.074	ρ = −0.201	ρ = −0.004
	*p* = <0.001 *	*p* = <0.001 *	*p* = 0.906	*p* = 0.366	*p* = 0.177	*p* = <0.001 *	*p* = 0.938

* Significant association (*p* < 0.05). HDL-C high-density lipoprotein cholesterol; HDL-C low-density lipoprotein cholesterol; WBC white blood cells. Warmer tones (red, orange, and yellow) and cooler tone (green) indicate positive and negative association, respectively. The stronger color represents higher level of association in both directions.

## Data Availability

Data are unavailable due to privacy or ethical restrictions.
